# *Orthoflavivirus omskense* NS1 Protein Induces Microvascular Endothelial Permeability In Vitro

**DOI:** 10.3390/v17070923

**Published:** 2025-06-28

**Authors:** Bogdana I. Kravchuk, Andrey L. Matveev, Andrey A. Kechin, Alena O. Stepanova, Lyudmila A. Emelyanova, Sargis M. Khachatryan, Nina V. Tikunova, Yana A. Khlusevich

**Affiliations:** 1 Institute of Chemical Biology and Fundamental Medicine Siberian Branch of Russian Academy of Sciences, 630090 Novosibirsk, Russia; semali328@gmail.com (B.I.K.); guterus@gmail.com (A.L.M.); a.a.kechin@gmail.com (A.A.K.); alena.o.lebedeva@gmail.com (A.O.S.); mila.kuharenko@mail.ru (L.A.E.); tikunova@niboch.nsc.ru (N.V.T.); 2 State Budgetary Healthcare Institution of the Novosibirsk Region “State Novosibirsk Regional Clinical Hospital”, 630087 Novosibirsk, Russia; sargis-k@yandex.ru

**Keywords:** OHFV, Omsk hemorrhagic fever virus, *Orthoflavivirus omskense* NS1 protein, hyperpermeability, endothelial dysfunction, TNF signaling pathway, vascular permeability regulators

## Abstract

*Orthoflavivirus omskense* (Omsk hemorrhagic fever virus, OHFV) is a tick-borne flavivirus that causes Omsk hemorrhagic fever (OHF), a severe zoonotic disease endemic to Western Siberia. Despite the fact that the role of NS1 proteins of various mosquito-borne flaviviruses in pathogenesis was investigated and their ability to affect human endothelial permeability was shown, the role of the NS1 protein of OHFV in pathogenesis is unstudied. In this work, the ability of OHFV NS1 to induce human endothelial permeability was investigated for the first time. It was shown that recombinant OHFV NS1 produced in eucaryotic cells directly affects both human lung microvascular endothelial cells (HLMVEC) and human umbilical vein endothelial cells (HUVEC) in vitro. RNAseq of endothelial cells treated with OHFV NS1 indicated that OHFV NS1 enhances the expression of genes associated with cellular stress responses, vascular signaling, and cell–cell junction regulation, resulting in a nonspecific increase in the endothelial permeability of various vessels. These results suggest that the NS1 protein may contribute to OHFV pathogenesis by disrupting endothelial barrier function and promoting vascular leakage, potentially playing a role in the hemorrhagic manifestations of Omsk hemorrhagic fever.

## 1. Introduction

*Orthoflavivirus omskense* (formerly known as Omsk hemorrhagic fever virus, OHFV) is a tick-borne flavivirus that causes Omsk hemorrhagic fever (OHF), a severe zoonotic disease, which is endemic to Western Siberia of the Russian Federation [1]. OHFV is most frequently transmitted to humans after the bite of ticks of the genera *Dermacentor* and *Ixodes*. Humans can also become infected through contact with infected muskrats (*Ondatra zibethicus*) [2,3]. During the 20th century, several OHF outbreaks were reported in the former USSR; however, this infection was found only in Western Siberia [4]. The most severe outbreak was reported from 1946 to 1958, during which more than 1000 cases of Omsk hemorrhagic fever infection were detected [5]. In this century, no cases of OHF have been observed until 2022 [4]. In 2022, OHF was detected in the Republic of Kazakhstan, which was the first case of the infection registered outside the Russian Federation. OHFV was detected in the cerebrospinal fluid of a patient who died of encephalitis of unknown etiology in Almaty city, located 1000 km from the nearest region, where OHFV had previously been reported [6].

The origin of OHFV is believed to be associated with a host-jumping event, when the virus transitioned from its primary vector, the *Ixodes persulcatus* tick, to a new host, the muskrat [3]. This host shift is thought to have occurred between 1931 and 1947, coinciding with the introduction of muskrats into Western Siberia [5,7]. The appearance of muskrats in this region created an ecological niche that allowed OHFV to adapt and establish itself as a distinct viral entity. Phylogenetic studies suggest that OHFV evolved from the tick-borne encephalitis virus (TBEV), with the virus undergoing adaptive amino acid substitutions in its E protein, which facilitated its transition to the muskrat host [1,3,8]. This adaptation enabled OHFV to utilize the muskrat as both a reservoir and an amplifying host, leading to its establishment in Western Siberia [7].

Currently, the primary natural reservoir of OHFV is the muskrat, which plays a crucial role in maintaining the virus in the environment, as they are highly susceptible to infection and can experience fatal epizootics [3,9]. The virus is maintained within muskrat populations through metaxenosis, a process where the virus is transmitted between different host species. In addition to muskrats, other small mammals and ticks may also contribute to the maintenance and circulation of OHFV in natural foci. Ticks, particularly *Dermacentor pictus* and *Ixodes persulcatus*, serve as both vectors and reservoirs of the virus. The virus can be transmitted vertically (from parent to offspring) and horizontally (between ticks and hosts) within tick populations [2].

Omsk hemorrhagic fever is characterized by high fever, hemorrhagic manifestations, and vascular dysfunction, leading to significant morbidity [1]. The incubation period of OHF lasts an average of 3–7 days. In all cases, the disease is characterized by continuous fever with a temperature of 39 °C–40 °C with the following symptoms: cough; headache; muscle aches; diarrhea; abdominal pain; rehydration; bleeding from the nose, mouth, and uterus; and skin hemorrhages. Fever may be accompanied by chills that last 8–15 days. With the course of the disease, primary hemorrhagic complications progress, and in very severe cases, there are gastrointestinal and pulmonary bleeding [4]. Usually, the duration of the disease is 1–2 weeks, after which 50–70% of patients recover without any complications. In 30–50% of cases, the second phase of the disease occurs; it lasts 5–14 days and is characterized by high fever and symptoms of diffuse encephalitis, such as continuous headache and meningitis. Bruises appear on the skin at pressure or injection sites. In addition, the lungs and kidneys may be affected, and bronchitis and pneumonia can appear. Chronic forms of OHF in humans have not been reported. In children, meningitis has been registered in 41% of cases [1].

The OHFV genome is a positive-sense single-stranded RNA of ~10.8 kb in length, with the open reading frame (ORF) approximately 10,200 bases [7]. OHFV ORF is flanked by the untranslated regions (UTRs). The 5′ untranslated region of OHFV contains a 5′ cap and has a sequence of about 30 nucleotides, which is not typical for other tick-borne flaviviruses [10]. OHFV ORF encodes a polyprotein that undergoes post-translational processing by viral and cellular proteases and consists of three structural (C, prM, E) and seven nonstructural proteins (NS1, NS2A, NS2B, NS3, NS4A, NS4B, and NS5) [11].

Nonstructural flavivirus protein 1 (NS1) is a conserved protein of ~352 amino acid residues in length with a molecular mass ranging from 46 kDa to 55 kDa depending on glycosylation status [12,13]. NS1 is the only flaviviral nonstructural protein secreted by infected cells [14]. It is known that NS1 of mosquito-borne flaviviruses plays an important role in the viral pathogenesis of hemorrhagic fevers caused by the viruses, particularly West Nile and dengue viruses (WNV and DENV) [15,16]. The ability of this protein to affect the endothelial permeability formed by the endothelium of various tissues has been demonstrated [15,17]. NS1-mediated vascular leakage has been extensively studied in mosquito-borne flaviviruses, namely DENV, Zika virus, Japanese encephalitis virus (JEV), and WNV [18,19]. In the case of DENV, NS1 disrupts endothelial permeability by interacting with the surface of endothelial cells and triggering the degradation of the endothelial glycocalyx, leading to a loss of barrier function. Furthermore, NS1 activates Toll-like receptor 4 (TLR4)-dependent inflammatory signalling, resulting in the production of cytokines such as TNF-α and IL-6, which in turn contribute to vascular leakage. Moreover, NS1 has been demonstrated to downregulate tight junction proteins, including ZO-1 and claudin-5, thereby compromising cell–cell junction integrity. Analogous effects have been reported for NS1 proteins of ZIKV and JEV, underscoring a conserved mechanism among flaviviruses. These findings emphasise the importance of NS1 in modulating endothelial function and underscore the relevance of studying OHFV NS1. Recently, the ability of the NS1 protein of TBEV to affect the permeability of human lung microvascular endothelial cells (HLMVECs) has been reported [20]. The pathogenesis of OHF and the role of the OHFV NS1 protein in it remain poorly understood.

Endothelial cells form a crucial barrier that regulates vascular integrity and permeability [21]. Disruption of this barrier could contribute to hemorrhagic symptoms observed in OHF. In this study, the ability of the OHFV NS1 protein to affect the microvascular endothelial permeability in various endothelial cell types was investigated. The ability of this protein to increase the permeability of endothelium-derived human lung microvascular endothelial cells (HLMVEC) and human umbilical vein endothelial cells was demonstrated using the solute flux assay and TEER. Using RNAseq, we found increased mRNA levels of genes associated with cellular stress responses, vascular signaling, and cell–cell junction regulation in OHFV NS1-treated HLMVECs.

## 2. Materials and Methods

### 2.1. Sera and Mammalian Cells

The plasmid pET32a-OHFV_NS1-sof, the plasmid pSB, *Escherichia coli* XL1-Blue cells, and CHO-s cells were obtained from the Collection of Extremophile Microorganisms and Type Cultures of ICBFM SB RAS. Endothelial cells HLMVEC were kindly provided by dr. Andrey Markov ICBFM SB RAS. Endothelial cells HUVEC were kindly provided by dr. Sargis Khachatryan, State Novosibirsk Regional Clinical Hospital. TBEV-positive sera were obtained from the Collection of Extremophile Microorganisms and Type Cultures of ICBFM SB RAS.

CHO-s cells were cultured using CD FortiCHO medium (Thermo Fisher Scientific, Waltham, MA, USA), supplemented with 2 mM GlutaMax I (Thermo Fisher Scientific, Waltham, MA, USA) and 1-x antibiotic antimycotic solution (Thermo Fisher Scientific, Waltham, MA, USA) in CO_2_-shaker at 150 rpm, 8% CO_2_ and 37 °C.

HLMVEC and HUVEC were cultured using EndoGRO-MV Complete Culture Media (Sigma Aldrich, St. Louis, MO, USA) in a 24-well plate treated with Collagen, Type I from rat tail (Sigma Aldrich, St. Louis, MO, USA).

### 2.2. Phylogenetic Analysis of NS1 Amino Acid Sequences

Amino acid sequences of the nonstructural protein 1 (NS1) from selected flaviviruses were obtained from the GenBank database. Multiple sequence alignment was carried out using the MUSCLE algorithm implemented in MEGA 12. A phylogenetic tree was inferred using the Maximum Likelihood method in MEGA 12, applying the LG model with a discrete Gamma distribution (+G) to account for rate variation among sites and allowing for a proportion of invariant sites (+I). The reliability of the inferred tree topology was assessed using 1000 bootstrap replicates. The final tree was visualized using the integrated tree viewer in MEGA and manually annotated for clarity. OHFV NS1 protein glycosylation sites were predicted using the NetNglyc 1.0 server, which predicts N-linked glycosylation sites in human proteins [22].

### 2.3. Construction of a Plasmid Encoding the NS1 OHFV Protein

To produce recombinant OHFV NS1 protein, the plasmid pSB-OHFV_NS1 was used. In this expression plasmid, the NS1 gene is located directly downstream of the human albumin gene leader sequence and upstream of the 6His tag coding sequence in the same ORF. The plasmid pSB-OHFV_NS1 contains the transposon inverted terminal repeats (ITRs) of the Sleeping Beauty transposase, the gene encoding puromycin N-acetyltransferase, and the gene encoding green fluorescent protein (GFP) (Figure S1).

The OHFV NS1 protein gene was amplified by PCR using primers Start_NS1_OHFV_56_pSB 5′-CCGTTGATATCGACGTTGGATGTGCTGTGGACACTGA-3′ and End_NS1_ OHFV_56_pSB 5′-CCGTTGGATCCGTGGTGATGGTGATGGTGAGCCACCACCATCGAGCGCAC-3′ and the pET32a-OHFV_NS1 as a matrix [23]. The expression plasmid pSB and the PCR fragment were then cleaved by restriction endonucleases *Eco*RV and *Bam*HI (Sibenzyme, Novosibirsk, Russia) and combined in a ligation reaction. *E. coli* XL1-Blue cells (recA1, endA1, gyrA96, thi, hsdR17(rK−, mK+), supE44, relA1, lac, [F′, proAB+, laclqZΔM15, Tn10(Tetr)]) were transformed with the resulting ligation product and seeded on LB-agar with ampicillin at a dose of 50 μg/mL and cultured. Individual colonies of *E. coli* cells containing plasmid pSB-OHFV_NS1 were screened by PCR using the same primers. PCR amplification conditions were as follows: 5 min at 95 °C, followed by 30 cycles of 30 s at 95 °C, 20 s at 56 °C, 1.5 min at 72 °C, and a final elongation of 6 min at 72 °C. The obtained PCR products were assessed by electrophoresis in 1% agarose gel. The accuracy of the insertion of the gene encoding the NS1 OHFV protein was confirmed by Sanger sequencing using primers NS1_SEQ_55U 5′-ACCAGAGTGATCGAGGCTGGGG-3′ and NS1_SEQ_55L 5′-CAGCGACGTAATCCCCCGTATG-3`. The resulting plasmid was pSB-OHFV_NS1, which encodes an OHFV NS1 protein with a His-tag at the C-terminus.

### 2.4. OHFV NS1 Protein Production and Purification

The suspension CHO-s cells were grown in a 125 mL Erlenmeyer Flask until the cell density reached 2 × 10^6^ cells/mL. Then, cells were co-transfected with the obtained plasmid pSB-OHFV_NS1 and pSB100x, that contained gene encoded sleeping beauty transposase gene was encoded using PeiPRO transfection reagent (Polyplus, Strasbourg, France). The efficacy of transfection was assessed using flow cytometry and confocal microscopy by evaluating the signal level of GFP in transfected cells 24 h after transfection. Forty-eight hours after transfection, GFP-positive cells were sorted using a cell sorter (SH800, Sony Biotechnology Inc., San Jose, CA, USA) into a 24-well plate containing selective media (CD FortiCHO, 2 mM glutamine, 1x solution antibiotic–antimycotic, 10 µg/mL puromycin). Selective media were replaced every 3–4 days with fresh media.

The OHFV NS1 protein was purified from the culture medium using metal-chelate chromatography on Ni-NTA agarose (Qiagen, Hamburg, Germany) according to the manufacturer’s instructions. Purified OHFV NS1 protein was dialyzed to phosphate buffer solution (PBS) and concentrated using Amicon centrifuge concentrators with a cutoff of 30 kDA to a concentration of 1 mg/mL. Purified OHFV NS1 was sterilized using a 0.22 μm syringe filter and stored at +4 C.

To confirm the oligomeric nature of the protein, its hydrodynamic radius was measured using a Zetasizer Nano (Malvern instrument, Malvern, Worcestershire, UK). The molecular weight was then calculated from this radius using an online converter (https://fluidic.com/molecular-weight-to-hydrodynamic-radius-converter/, accessed date 21 June 2025). The hydrodynamic radius of the NS1 protein was found to be approximately 6 nm. This corresponds to a molecular weight of over 300 kDa, indicating that the protein is present in the solution in the form of a hexamer (Figure S2).

### 2.5. ELISA and Western Blot Analysis

For indirect ELISA, 1 μg/mL of purified recombinant OHFV NS1 protein was adsorbed into the wells of 96-well polystyrene plates (Greiner, Kremsmünster, Austria), then the nonspecific binding sites were blocked with 5% skim milk solution. Five-fold serial dilution of two immune ascites fluids (anti-OHFV IAF) that were collected from mice independently infected with OHFV strain P-15-2213 and OHFV strain Oz-31_Kd_10866 (started dilution 1:500) or monoclonal antibodies NS1-1.3, NS1-1.6, NS1-2.299, NS1-2.290, and NS1-2.44, specifically recognizing native and recombinant TBEV NS1 at a concentration of 10 μg/mL, were added to the wells [24,25]. Then, wells were incubated with Anti-Mouse IgG (Fc specific) HRP conjugated antibody produced in rabbit (Biosan, Novosibirsk, Russia). Immune complexes were detected using 3,3′,5,5′-tetramethylbenzidine (TMB, Applichem, Solon, OH, USA). Optical density was assessed at a wavelength of 450 nm using a microplate reader iMark (Bio-Rad, Hercules, CA, USA).

The purified recombinant OHFV NS1 protein was fractionated using 12.5% PAGE and then transferred to a nitrocellulose membrane (Bio-Rad, Hercules, CA, USA). The nonspecific binding sites were blocked with 3% bovine serum albumin solution (BSA, Amresco, Solon, OH, USA). The membrane was incubated with immune ascitic fluids obtained from OHFV-infected mice at a dilution of 1:5000. The membrane was then incubated with Anti-Mouse IgG (Fc specific)–peroxidase antibody produced in rabbit (Biosan, Novosibirsk, Russia). Immune complexes were detected using 4-chloro-1-naphthol (Applichem, Darmstadt, Germany). Samples pre-incubated with antibodies against NS1 were used as negative controls.

### 2.6. Solute Flux Assay

To assess the effect of OHFV NS1 protein on the transit of macromolecules through the human epithelial cell monolayer, HLMVEC or HUVEC cells (60,000 or 80,000 cells/insert, respectively) were grown on collagen-coated PC Membrane Cell Culture Inserts for 24-well plates with a pore size of 0.4 μm and a diameter of 6.5 mm in the volume of 300 μL of EndoGRO-MV Complete Culture Media per insert. Each insert was transferred into a well of a 24-well plate containing 1.2 mL of EndoGRO-MV Complete Culture Media. One day before the experiment, 50% of the medium was replaced with fresh EndoGRO-MV Complete Culture Media. Recombinant OHFV NS1 protein at a concentration of 10 μg/mL was then added to an insert containing a monolayer of cells. Five hours after the start of the experiment, streptavidin conjugated with horseradish peroxidase (Sigma) was added to the insert at a final concentration of 100 ng/mL and incubated for 20 min at 37 °C. The inserts were removed, and 100 μL of culture fluid was collected from each well (lower chamber) of a 24-well plate. Horseradish peroxidase activity was determined using tetramethyl benzidine, and the concentration of biotin-conjugated horseradish peroxidase from the insert into a well of a 24-well plate was determined by plotting a standard horseradish peroxidase curve. The signal was measured using an iMark plate reader (Bio-Rad). TNF-α (100 ng/mL) was used as a positive control, and untreated cell monolayers were used as a negative control.

### 2.7. Endothelial Permeability Test by Measuring Trans-Endothelial Electrical Resistance (TEER)

The permeability of endothelial cells treated with recombinant OHFV NS1 proteins was assessed by measuring TEER of these cells. A total of 50,000 cells for HUVEC and HLMVEC were seeded in the PC Membrane Cell Culture Inserts for 24-well plates with a pore size of 0.4 μm and a diameter of 6.5 mm (Wuxi NEST Biotechnology Co., Ltd., Wuxi, China), in the volume of 300 μL of EndoGRO-MV Complete Culture Media per insert. Each insert was transferred into a well of a 24-well plate containing 1.2 mL of EndoGRO-MV Complete Culture Media. Twenty-four-well plates with inserts were incubated at 37 °C and 5% CO_2_ until TEER ranges of 150–180 ohm (Ω) were reached. Recombinant OHFV NS1 protein at a final concentration of 10 μg/mL was then added to an insert containing a monolayer of cells. TEER values were measured at consecutive 1 h time points after the treatment of recombinant protein using an EVOM3 epithelial Volt/Ohm (TEER) Meter (World Precision Instruments, Sarasota, FL, USA). TEER values were measured at consecutive 1 h time points after addition of test proteins using an epithelial volt-ohmmeter (EVOM) with “wand” electrodes (World Precision Instruments). Endothelial permeability was calculated as relative TEER, using the following formula: (Ω treated endothelial cells − Ω medium)/(Ω untreated endothelial cells − Ω medium).

### 2.8. RNA-seq and Data Analysis

A total of 100,000 cells of HLMVEC were seeded in the wells of a 12-well plate in a volume of 1 mL of EndoGRO-MV Complete Culture Media. The 12-well plate was incubated at 37 °C and 5% CO_2_ until a monolayer of HLMVEC cells was formed. The cells were treated with 50 μL per well recombinant OHFV NS1 protein in the concentration of 200 μg/mL in PBS or only 50 μL PBS and incubated for 3 h at 37 °C and 5% CO_2_. After the culture media was removed, 1 mL of TRIzol™ Reagent (Thermo Fisher Scientific, Waltham, MA, USA) was added to each well to lyse cells. Then, cell lysates were frozen and stored at −70 °C. RNA isolation and RNA seq were carried out in the laboratory of Genomed LLC (Moscow, Russia).

Four biological samples were used for sequencing (n = 2 OHFV NS1-treated and n = 2 PBS-treated controls). Total RNA was extracted using the RNeasy Mini Kit (Qiagen), and RNA integrity was assessed with the Agilent 2100 Bioanalyzer and Qubit fluorometer. Library preparation was performed using a poly(A)-enrichment and strand-specific protocol. Sequencing was performed on the DNBSEQ platform with 2 × 100 bp paired-end reads, generating approximately 40 million reads per sample.

Quality assessment of the raw FASTQ sequencing data was carried out using FastQC. Reads were aligned to the Ensembl mouse reference genome (GRCm39) using HISAT2, resulting in raw gene-level read counts for each sample. SAM files were converted to BAM format for efficient processing. Gene-level read counts were obtained with featureCounts, and the resulting count matrix was used as input for DESeq2 to identify differentially expressed genes.

### 2.9. Statistics

Statistically significant differences between the OHFV NS1-treated endothelium cells group and the OHFV NS1 non-treated endothelium cells group were evaluated by two-way ANOVA analysis using Dunnett’s test for multiple comparisons. The Statistica 10 software package (StatSoft Inc., Tulsa, OK, USA) was used to perform statistical analysis. Normality was assessed using the Shapiro–Wilk test, and statistical significance was set at α = 0.05.

## 3. Results

### 3.1. Phylogenetic Relationships of OHFV NS1 Protein Within the Tick-Borne Flavivirus Complex

**Figure 1:**
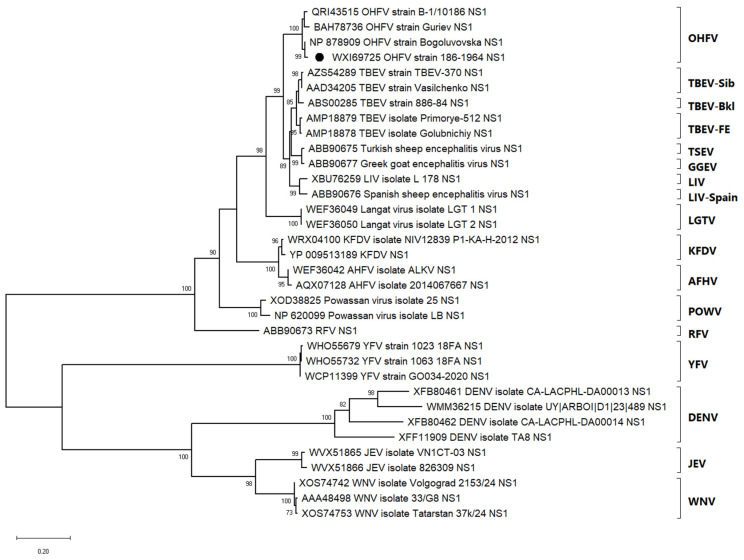
Maximum likelihood phylogenetic tree based on NS1 amino acid sequences of representative flaviviruses. The tree was generated using the LG+G+I substitution model with 1000 bootstrap replicates. Bootstrap values above 70% are shown next to the branches. Major flavivirus groups, including OHFV (Omsk hemorrhagic fever virus), TBEV (tick-borne encephalitis virus: Siberian, Baikalian, Far Eastern), LIV (Louping ill virus), AHFV (Alkhurma hemorrhagic fever virus), POWV (Powassan virus), DENV (Dengue virus), JEV (Japanese encephalitis virus), and WNV (West Nile virus), form distinct clades as indicated. The OHFV strain used in this study is marked with a black circle.

To establish possible links between the amino acid sequences of NS1 proteins of flaviviruses causing haemorrhagic symptoms, a phylogenetic analysis was performed. The maximum likelihood phylogenetic tree constructed from NS1 amino acid sequences using the LG+G+I model (Figure 1) reveals the evolutionary relationships among a broad range of flaviviruses. Sequence of NS1 protein, previously used to obtain the recombinant protein [25], clusters within the OHFV group, forming a monophyletic clade together with other OHFV strains (B-1/10186, Guriev, and Bogulovska), supported by a high bootstrap value of 100%. This cluster is clearly separated from the TBEV complex. The tree includes more distantly related tick-borne flaviviruses such as Kyasanur Forest disease virus (KFDV), Alkhurma hemorrhagic fever virus (AHFV), and Powassan virus (POWV), each forming well-supported individual clades. In the mosquito-borne flavivirus group, which branches off distinctly from the tick-borne lineage, DENV isolates cluster together with high support (bootstrap value = 100%), while Yellow fever virus (YFV), JEV, and WNV each form individual, well-supported clades.

It was predicted that the OHFV NS1 protein has three putative N-linked glycosylation sites at residues N85, N207, and N223.

The clear separation between tick-borne and mosquito-borne flaviviruses confirms the deep evolutionary divergence between these ecological groups. Altogether, the tree topology supports the taxonomic classification of flaviviruses and confirms the distinct genetic identity of OHFV while illustrating its close relationship with other members of the tick-borne flavivirus complex.

### 3.2. Production and Purification of OHFV NS1 Protein

**Figure 2:**
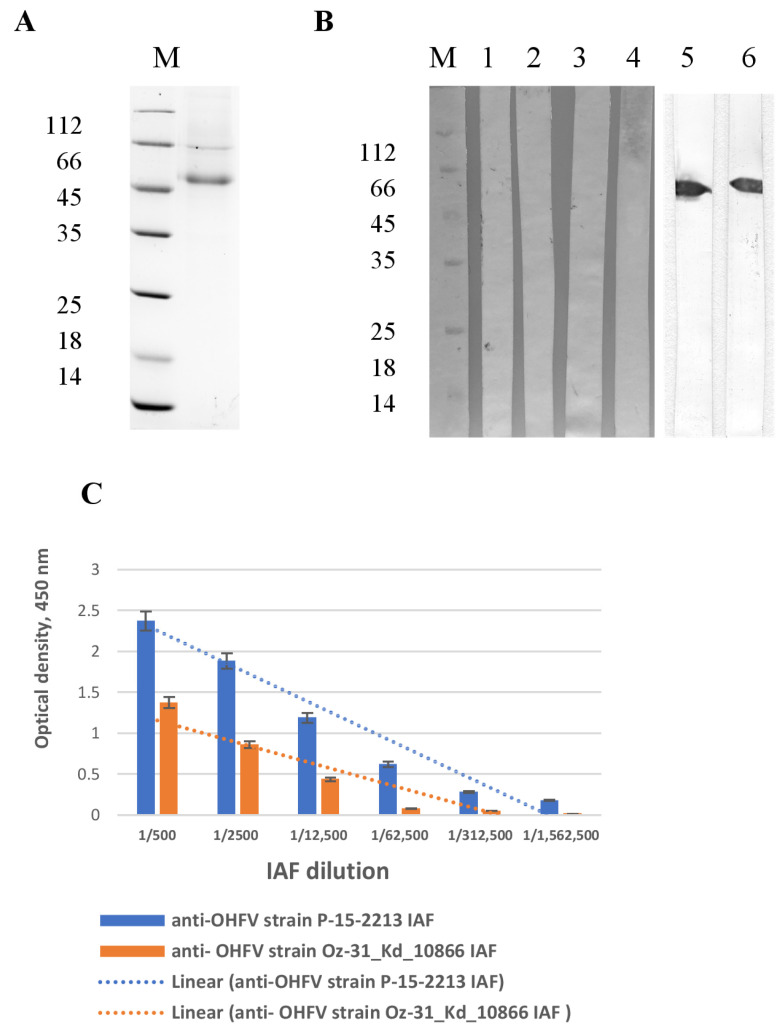
Characterization of recombinant OHFV NS1 protein purified from CHO-S cells. (**A**) SDS-PAGE of OHFV NS1. (**B**) Western blot analysis of the recombinant OHFV NS1 revealed by monoclonal antibodies against native TBEV NS1, namely NS1-1.3 (line 1), NS1-1.6 (line 2), NS1-2.299 (line 3), NS1-2.290 (line 4) or anti-OHFV strain P-15-2213 IAF (line 5), and anti-OHFV strain Oz-31_Kd_10866 IAF (line 6) at a dilution of 1:5000; M—marker of molecular mass in kilodaltons. (**C**) ELISA of serial dilution of immune ascetic fluids from mice infected with OHFV strains P-15-2213 and Oz-31_Kd_10866 to bind recombinant OHFV NS1.

The resulting plasmid pSB-OHFV_NS1 was transfected into CHO-s cells. Immobilized metal chelate affinity chromatography on Ni-NTA resin was used to purify the recombinant OHFV NS1 protein from the culture medium. The electrophoretic mobility of the purified OHFV NS1 protein was consistent with its theoretically predicted molecular mass of 52 kDa (Figure 2A). Homogeneity of purified OHFV NS1 protein assessed by PAGE was approximately 80%. A total of 15 mg of purified NS1 protein was harvested from 1 L of culture medium. The purified OHFV NS1 protein was concentrated to a concentration of 1 mg/mL in PBS, filtered through a 0.22 syringe filter, and stored at 4 °C. The hydrodynamic radius of the NS1 protein was assessed using dynamic light scattering to be approximately 6 nm. This corresponds to a molecular weight of over 300 kDa, indicating that the protein is present in the solution in the form of a hexamer.

### 3.3. Immunological Recognition and Antigenic Characterization of Recombinant OHFV NS1 Protein

To confirm that the recombinant protein had folded correctly, we investigated its immunological properties. The immunological properties and antigenic profile of the recombinant OHFV NS1 protein were evaluated by ELISA and Western blot analysis. Western blot analysis indicated that the anti-OHFV strain P-15-2213 IAF and the anti-OHFV strain Oz-31_Kd_10866 IAF revealed a band corresponding to CHO-derived OHFV NS1 protein (Figure 2B and Figure S3). None of the antibodies against native TBEV NS1 identified a band corresponding to the CHO-derived OHFV NS1 protein (Figure 2B). Thus, we obtained a protein with confirmed correct folding.

ELISA was used to test the binding of serial dilutions of two anti-OHFV IAFs to recombinant OHFV NS1. Antibodies present in both anti-OHFV IAFs bound recombinant OHFV NS1 proteins at a dilution of 1:100,000 for the anti-OHFV strain Oz-31_Kd_10866 IAF and 1:4,500,000 for the anti-OHFV strain P-15-2213 IAF (Figure 2C). These results confirm the antigenic integrity of the recombinant OHFV NS1, supporting its relevance for further functional studies, including the analysis of its impact on endothelial permeability.

### 3.4. Effect of Recombinant OHFV NS1 Protein on Human Endothelial Permeability In Vitro

**Figure 3:**
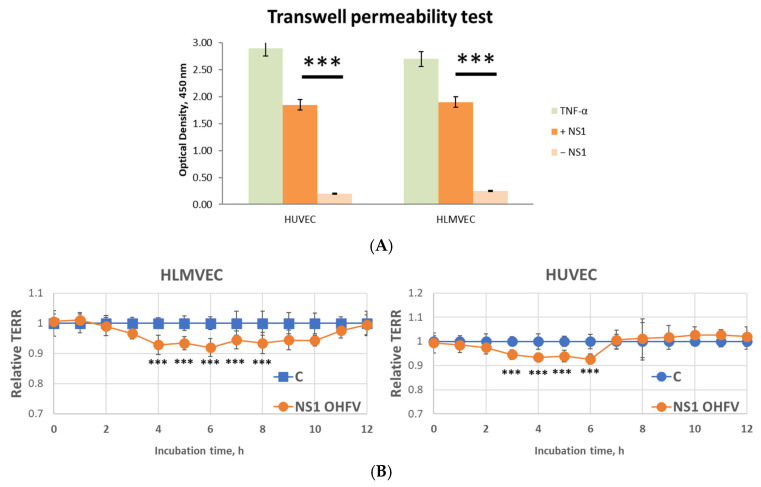
Evaluation of the effect of the OHFV NS1 protein on endothelial permeability at the indicated time points over 12 h. The permeability of human lung microvascular endothelial cells (HLMVEC) and human umbilical vein endothelial cells (HUVEC) was examined using (**A**) transwell permeability test; (**B**) EVOM3 epithelial Volt/Ohm (TEER) real-time transendothelial electrical resistance assay. The results of the assay are shown in relative units. The cell index of control cells (not treated with OHFV NS1) was taken as 100%. Statistically significant differences between the OHFV NS1-treated group and the OHFV NS1 non-treated group were evaluated by two-way ANOVA analysis using Dunnett’s test for multiple comparisons, with *** *p* < 0.01.

Two endothelial cell lines, HLMVEC and HUVEC, were used to evaluate the effect of OHFV NS1 protein on endothelial permeability. It was shown by solute flux assay that treatment of HLMVEC and HUVEC cells with OHFV NS1 protein led to an 8-fold and 7-fold increase in endothelial monolayer permeability, respectively, compared with the negative control during the first 5 min of the experiment (Figure 3A).

The effect of the OHFV NS1 protein on endothelial cell permeability was further tested using TEER measurements, and the results confirmed the solute flux assay data. For HUVEC cells, a 6.7% decrease in monolayer resistance was observed starting 3 h after treatment with OHFV NS1 protein compared to the control. The maximum decrease in resistance was reached in 6 h. The TEER of the cells returned to a plateau after 7 h. Although TEER dynamics differed between HUVEC and HLMVEC cells after NS1 treatment, no direct statistical comparison between these two cell types was performed. The changes in TEER of treated cells indicated an increase in the permeability of the endothelial layer formed by HUVECs after treatment with OHFV NS1 protein (Figure 3B).

The effects of the OHFV NS1 protein on the permeability of the HLMVEC line were more significant (Figure 3B). There was a 6.5% decrease in monolayer resistance starting 4 h after treatment with OHFV NS1 protein compared to the control. The maximum decrease in resistance was reached after 6 h. TEER of cells returned to a plateau after 12 h. RNA sequencing (RNA-seq) of HLMVECs treated with the OHFV NS1 protein was performed to determine the possible molecular mechanisms responsible for this process.

### 3.5. Transcription Profile of Human Lung Capillary Endothelial Cells Threated with OHFV NS1 Recombinant Protein

**Figure 4:**
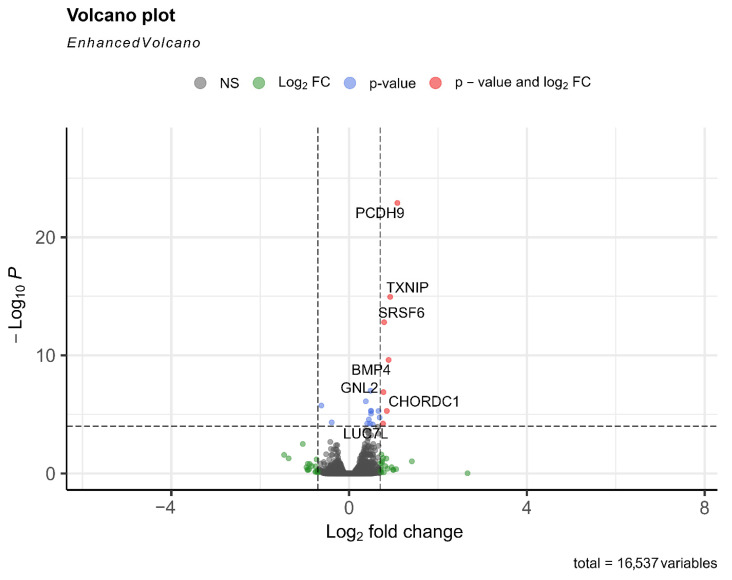
Volcano plot of differential gene expression analysis. The x-axis shows the Log_2_ fold change, and the y-axis displays the −Log_10_ *p*-value. Genes meeting both significance thresholds for fold change and *p*-value are highlighted in red. Selected significantly upregulated genes (PCDH9, TXNIP, SRSF6, BMP4, GNL2, CHORDC1, LUC7L) are annotated.

To identify differentially expressed genes (DEGs) between treated and control samples, we applied DESeq2 to the feature counts-derived matrix. A total of 16,537 genes were analyzed. The resulting volcano plot (Figure 4) highlights genes with significant expression changes, using thresholds of |log_2_ fold change| > 1 and adjusted *p*-value < 0.05. Key upregulated DEGs include TXNIP (a redox-responsive gene), SRSF6 (involved in RNA splicing), BMP4 (linked to vascular development), PCDH9 (a protocadherin involved in cell adhesion), GNL2 (Nucleolar GTP-binding protein 2), CHORDC1 (CHORD domain-containing protein 1), and LUC7L (Luc7-like). These transcriptional shifts suggest a coordinated activation of pathways related to cellular stress responses, vascular signaling, and cell–cell junction regulation.

**Figure 5:**
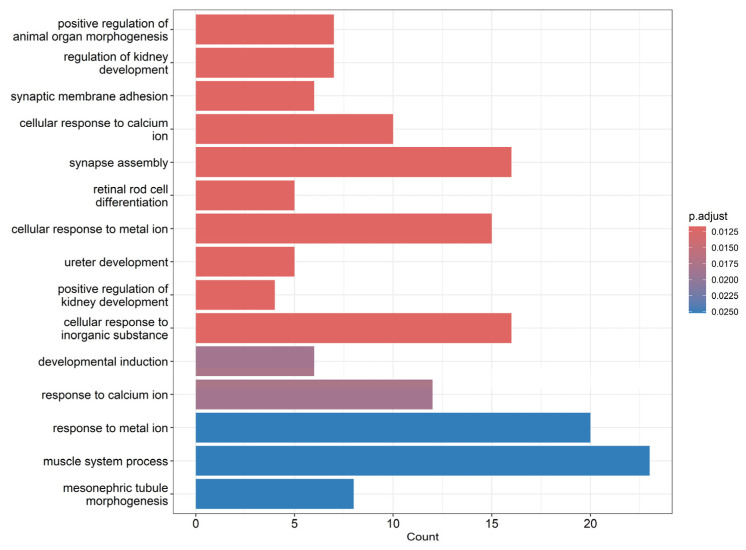
Bar plot representing the results of Gene Ontology (GO) enrichment analysis for biological processes. The x-axis indicates the number of genes associated with each GO term, while the y-axis lists the significantly enriched biological processes.

Gene Ontology (GO) enrichment analysis of the differentially expressed genes revealed significant alterations in biological processes linked to vascular function and integrity. Terms such as “cellular response to calcium ion” and “response to metal ion” suggest disruption of endothelial ion homeostasis, a known trigger of vascular permeability changes (Figure 5). The enrichment of “synapse assembly” and “neurovascular signaling” pathways may indicate endothelial crosstalk with neural components, relevant in the context of neuroinvasive flaviviruses. Processes like “muscle system process” and “mesonephric tubule morphogenesis” imply potential impacts on vascular smooth muscle and renal vasculature development. Collectively, these changes support the hypothesis that the OHFV NS1 protein induces endothelial dysfunction and tissue-specific vascular remodeling, contributing to increased microvascular permeability and possibly underlying the pathophysiological features of OHFV-associated disease.

## 4. Discussion

Endothelial cells form a crucial barrier that regulates vascular integrity and permeability [21,26]. Disruption of this barrier could contribute to hemorrhagic symptoms observed in OHF. Emerging evidence suggests that flavivirus NS1 proteins can interact with endothelial cells in a tissue-specific manner, leading to increased permeability and vascular dysfunction [17,27].

The close phylogenetic relationship between OHFV and TBEV supports the hypothesis that these viruses share a recent common ancestor. The phylogenetic positioning of OHFV suggests that it evolved within the tick-borne flavivirus lineage. Although OHFV is clinically classified as a hemorrhagic fever, phylogenetic analyses based on NS1 protein sequences consistently place it in close evolutionary proximity to members of the TBEV complex, such as TBEV, Louping ill virus (LIV), and Turkish sheep encephalitis virus (TSEV). In contrast, other hemorrhagic tick-borne flaviviruses, namely KFDV and AHFV, cluster separately within the tick-borne group. This phylogenetic distinction suggests that, despite overlapping clinical features, the hemorrhagic phenotype in OHFV likely emerged independently from that of KFDV and AHFV. Taken together, these facts support a model of convergent evolution, in which hemorrhagic manifestations evolved in parallel within distinct tick-borne flavivirus lineages through separate ecological and host-driven adaptations, rather than being inherited from a common hemorrhagic ancestor.

Phylogenetic analysis of the amino acid sequence of the OHFV NS1 protein showed that it is clustered distantly from the NS1 proteins of flaviviruses causing hemorrhagic fevers and carried by mosquitoes (DENV, YFV) with an identity level of less than 50% and ticks (KDFV and AHFV) with an identity level of less than 75%. OHFV NS1 is phylogenetically closest to TBEV NS1 of the Siberian subtype (identity level more than 85%). According to one version, OHFV originated from TBEV [3]. Notably, a single outbreak of TBEV of the Far Eastern subtype with hemorrhagic form was detected in 1999 in the Novosibirsk region [28], and no new cases of tick-borne encephalitis with hemorrhagic form were reported. This outbreak was likely caused by an infection consisting of TBEV combined with an undetected pathogen.

Bioinformatics analysis showed that the OHFV NS1 protein has a theoretical N-linked glycosylation pattern similar to TBEV NS1. OHFV NS1 has three putative N-linked glycosylation sites at residues N85, N207, and N223. Most members of the genus Flavivirus have two N-linked glycosylation sites, N130 and N207, including JEV, ZIKV, and all four serotypes of DENV [29]. Some representatives of mosquito-borne flaviviruses, such as WNV, SLEV, and MVEV, have a third glycosylation site located at residue N175 [30]. The n-linked glycosylation of the NS1 protein of flaviviruses likely does not affect the appearance of hemorrhagic symptoms.

The predicted structure of OHFV NS1 was compared with the predicted structure of TBEV NS1, showing their structural similarities [25]. Nevertheless, differences in the antigenic profiles of OHFV NS1 and TBEV NS1 have been proved previously [25] as monoclonal antibodies against TBEV NS1 and sera from volunteers with confirmed TBE did not bind OHFV NS1.

The ability of TBEV NS1 to influence endothelial permeability was previously investigated. This study showed that TBEV NS1 demonstrates tissue specificity to endothelial cells. TBEV NS1 increased endothelial permeability in HLMVEC cells but not HUVECs. RNAseq indicated that treatment of HLMVEC cells with TBEV NS1 activated the TNF-signaling pathway [20].

In contrast to the TBEV NS1 protein, the OHFV NS1 protein did not demonstrate tissue-specificity to endothelial cells. OHFV NS1 increased the permeability of the endothelium formed by both HLMVEC and HUVEC. RNAseq results showed that treatment of HLMVEC cells with OHFV NS1 activated the expression of GNL2 (Nucleolar GTP-binding protein 2), CHORDC1 (CHORD domain-containing protein 1), LUC7L (Luc7-like), TXNIP (a redox-responsive gene), SRSF6 (involved in RNA splicing), BMP4 (linked to vascular development), and PCDH9 (a protocadherin involved in cell adhesion). These transcriptional shifts suggest a coordinated activation of pathways related to cellular stress responses, vascular signaling, and cell–cell junction regulation.

These transcriptional shifts indicate activation of pathways related to cellular stress, vascular signaling, and junctional integrity. TXNIP is a redox-sensitive regulator linked to oxidative stress and endothelial inflammation. BMP4, a TGF-β superfamily cytokine, is associated with vascular remodeling and dysfunction. PCDH9, a cadherin family protein, contributes to cell–cell adhesion and may influence junctional stability. Upregulation of splicing factors SRSF6 and LUC7L suggests changes in RNA processing that could affect proteins involved in endothelial barrier function. CHORDC1, a stress-related co-chaperone, and GNL2, involved in ribosome biogenesis and cell proliferation, may reflect a cellular response to NS1-induced stress or activation.

NS1 proteins from various mosquito-borne flaviviruses have been shown to increase endothelial cell permeability in tissues associated with each flavivirus disease’s viral tropism. Specifically, NS1 from DENV causes systemic disease, which causes hyperpermeability in endothelial cells of the lung, skin, umbilical vein, brain, and liver [31]. NS1 from ZIKV, which affects the placenta and developing brain, causes hyperpermeability only in endothelial cells of the umbilical vein and brain [19]. NS1 from YFV, which is systemic but causes predominantly hepatic lesions, had the strongest effect on hepatic endothelial cells’ permeability, with a slight increase in permeability in pulmonary endothelial cells [17].

Although OHFV and TBEV are closely related species, the clinical manifestations of infection with these viruses differ significantly. Like dengue fever, OHF is a systemic disease associated with nasal, oral, uterine, and cutaneous hemorrhages. Both OHFV NS1 and DENV NS1 can likely cause endothelial permeability disorders in a wide range of tissues and organs, promoting viral penetration into these organs and being one of the factors in the development of hemorrhagic complications. However, TBE manifests as meningitis, encephalitis, or meningoencephalitis without hemorrhagic complications. One of the probable reasons for such differences in pathogenesis is the tissue specificity of TBEV NS1 to the endothelium.

## 5. Conclusions

NS1 protein is not the only factor determining hemorrhagic manifestations in flavivirus hemorrhagic fevers, but it is likely to play an important role in pathogenesis, contributing to changes in vascular permeability that ameliorate the penetration of flaviviruses into various organs and tissues. Although similar mechanisms remain poorly characterized for OHFV, available data suggest that NS1 may similarly contribute to vascular pathology in this context. Our findings provide new insights into the mechanisms of OHFV-induced vascular pathology and offer a foundation for future research on targeted interventions against flavivirus-mediated endothelial dysfunction and pathogenesis of hemorrhagic fevers.

## Data Availability

Data are contained within the article and Supplementary Materials.

## Supplementary Materials

The following supporting information can be downloaded at: https://www.mdpi.com/article/10.3390/v17070923/s1, Figure S1: Scheme of plasmid map pSB-OHFV_NS1. The pCAG promoter, Albumin leader, OHFV NS1 protein genes, and puromycin resistance gene fused to GFP protein via p2a-peptide were labeled. Figure S2. Characterization of oligomeric of OHFV NS1 form by dynamic light scattering, using Zetasizer Nano (Malvern instruments). It was identified that average hydrodynamic radius was 6.095 ± 2.084 nm. Figure S3. Western blot analysis of the recombinant OHFV NS1 revealed by anti-OHFV strain P-15-2213 IAF (line 1) and anti-OHFV strain Oz-31_Kd_10866 IAF (line2) at a dilution of 1:500. The red arrow shows the monomeric form and the upper black arrow shows the dimeric form.

## Abbreviations

The following abbreviations are used in this manuscript:

OHFV	Omsk hemorrhagic fever virus
TBEV	tick-borne encephalitis virus
OHF	Omsk hemorrhagic fever
ORF	open reading frame
NS1	Nonstructural flavivirus protein 1
WNV	West Nile virus
DENV	dengue virus
JEV	Japanese encephalitis virus
HLMVEC	human lung microvascular endothelial cells
TEER	transepithelial electrical resistance
