# Supplementary material for: Orthoflavivirus omskense NS1 Protein Induces Microvascular Endothelial Permeability In Vitro
Source: Viruses. 2025 Jun 28;17(7):923. doi: 10.3390/v17070923 (PMC12300036; doi:10.3390/v17070923)
Supplement: Supplementary file 1 [file viruses-17-00923-s001.zip › viruses-3657952-supplementary.pdf]

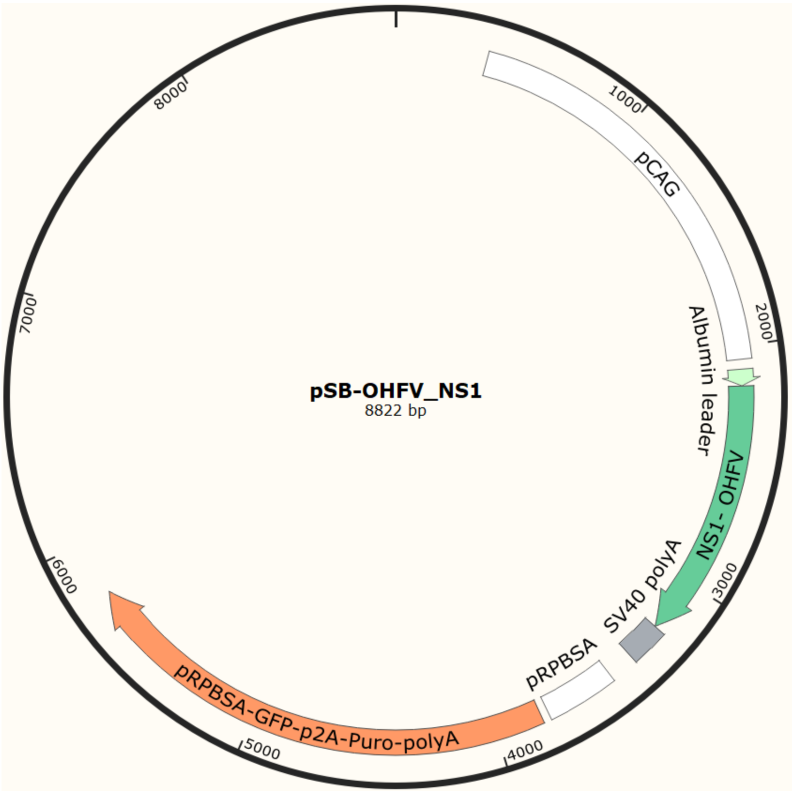

Figure S1. Scheme of plasmid map pSB-OHFV_NS1. The pCAG promoter, Albumin leader, OHFV NS1 protein genes, and puromycin resistance gene fused to GFP protein via p2a-peptide were labeled.

**SOP Name:**	mansettings.nano		
**File Name:**	17.06.2025-Матвеев	**Dispersant Name:**	ICN PBS Tablets
**Record Number:**	1	**Dispersant RI:**	1,330
**Material RI:**	1,45	**Viscosity (cP):**	0,8882
**Material Absorbtion:**	0,001	**Measurement Date and Time:**	17 июня 2025 г. 11:49:04

**Temperature (°C):**	25,0	**Duration Used (s):**	70
**Count Rate (kcps):**	209,4	**Measurement Position (mm):**	3,00
**Cell Description:**	Disposable micro cuvette (40µl)	**Attenuator:**	10

			**Size (d.nm):**	**% Number:**	**St Dev (d.nm):**
**Z-Average (d.nm):**	75,13	**Peak 1:**	12,19	100,0	4,168
**PdI:**	0,723	**Peak 2:**	0,000	0,0	0,000
**Intercept:**	0,912	**Peak 3:**	0,000	0,0	0,000

Result quality : **Refer to quality report**

Figure S2. Characterization of oligomeric of OHFV NS1 form by dynamic light scattering, using Zetasizer Nano (Malvern instruments). It was identified that average hydrodynamic radius was 6.095 ± 2.084 nm.

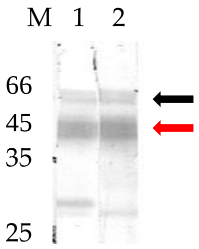

Figure S3. Western blot analysis of the recombinant OHFV NS1 revealed by anti-OHFV strain P-15-2213 IAF (line 1) and anti-OHFV strain Oz-31_Kd_10866 IAF (line2) at a dilution of 1:500. The red arrow shows the monomeric form and the upper black arrow shows the dimeric form.
